# Visual pursuit response in the severe disorder of consciousness: modulation by the central autonomic system and a predictive model

**DOI:** 10.1186/1471-2377-13-164

**Published:** 2013-11-06

**Authors:** Francesco Riganello, Maria D Cortese, Giuliano Dolce, Walter G Sannita

**Affiliations:** 1 S. Anna Institute and RAN (Research in Advanced Rehabilitation), Crotone, Italy; 2 Department of Neuroscience, Ophthalmology and Genetics, University of Genova, 3, Largo P. Daneo, 16132 Genova, Italy; 3 Department of Psychiatry, State University of New York, Stony Brook, NY, USA

**Keywords:** Disorder of consciousness, Visual pursuit response, Vegetative state, Minimally conscious state, Central autonomic system, Heart rate variability, Support vector machine

## Abstract

**Background:**

A visual pursuit response is reportedly observed in ~20-30% of subjects in vegetative state (VS/UWS) and predicts better outcome; it is a key marker of evolution into the minimally conscious state (MCS). The probability of observing a positive response, however, has proven variable during the day, with comparable timing of the minima and maxima in VS/UWS and MCS. We verified if measures of sympathetic/parasympathetic balance are possible independent variables on which the occurrence of a pursuit response could depend and be predicted.

**Methods:**

Fourteen subjects in VS/UWS and sixteen in MCS for more than one year were studied. A mirror was used to test the pursuit response for a total 231 useful trials. Non-invasive measures of the sympathetic/parasympathetic functional state (Heart rate variability descriptors nuLF and peakLF) used in the study of responsiveness in VS/UWS and MCS subjects were recorded and processed by descriptive statistics and advanced Support Vector Machine (SVM).

**Results:**

A pursuit response was observed in 33% and 78.2% of subjects in VS or MCS, respectively. Incidence was higher at HRV nuLF values in the 20–60 range and peakLF values at 0.06-0.12 Hz (76.6%) and at nuLF values in the 10–60 range and peakLF values at 0.05-0.10 Hz (80.7%) in the VS and MCS, respectively. The SVM generated model confirmed the results in the training leave one out and 10 fold cross validation tests (81% and 81.4%).

**Conclusion:**

The pursuit response incidence depends to a relevant extent on the sympathetic/parasympathetic balance and autonomic functional state. Extensive monitoring appears advisable.

## Background

A visual pursuit response (the “*pursuit eye movement or sustained fixation that occurs in direct response to moving or salient stimuli”*) is reportedly observed in ~20-30% of the severely brain damaged subjects otherwise characterized by all clinical features of the vegetative state (VS; also referred to as the unresponsive wakefulness syndrome or UWS) [1] and predicts a favorable outcome with accuracy higher than 70%. The response is also a key marker of evolution into the minimally conscious state (MCS; a condition with evidence of partially recovered awareness of self or environment, in which it is observed in ~70-80% of subjects) and is a major item of the revised Coma Recovery Scale [1,2].

The response incidence in VS/UWS and MCS is thought to further blur boundaries between these conditions and to reduce diagnostic accuracy, with an estimated misdiagnosis rate up to 25-45% [3]. Multiple testing on subjects in VS/UWS or MCS (6 tests/subject/day) confirmed a varying incidence of the pursuit response during the day, with maxima at 10.30 am and 3.00 pm, no response at postprandial time, and an overall chance of observing it at least once per day of ~33% and ~62% in the VS/UWS and MCS, respectively [4]. The occurrence of a positive pursuit response thus appears conditional to changes in the functional brain state that may occur spontaneously or be induced by a variety of possible neuronal or non-neuronal factors, and may question the sign reliability and pathophysiological meaning.

Replicable changes in the sympathetic/parasympathetic functional state in response to simple emotional or complex sensory stimulus conditions have been described in VS/UWS and MCS subjects [5-7] for references. The observation is in the same line of evidence with neuroimaging studies documenting in these patients a residual responsiveness, *i.e.* stimulus- or condition-related regional brain activation [8,9]. Measures of the sympathetic/parasympathetic balance thus appear to be possible independent variables against which the occurrence of a pursuit response be tested. To this end, we have investigated in VS/UWS and MCS subjects the correlation between the presence/absence of a pursuit response and measures of heart rate variability (HRV). HRV (*i.e.* the heart rate fluctuation around the mean value over the time sample) is regarded today as a reliable index of the sympathetic/parasympathetic interplay and intrinsic influence on heart rate; it is deemed also applicable in the description of the brain and autonomic system interaction and functional organization in homeostasis and homeostatic responses both in awake subjects and in the severe disorder of consciousness [10-13].

## Methods

### Subjects

Fourteen subjects diagnosed as being in VS/UWS (10 males, age range: 27–73 yrs., mean: 45 ± 17 yrs.) and sixteen in MCS (9 males, age range: 33–77 yrs., mean: 55.2 ± 16.3 yrs.) for more than one year according to the current clinical criteria and established evaluation scales were studied [2]. Scores were lower than 25 at the Loewenstein Scale [14], 2 at the Level of Cognitive Function scale (LCF) [15] and higher than 21 at the Disability Rating Scale [16] in the VS/UWS; LCF scores were 3 for subjects in the MCS. The global score of the revised Coma Recovery Scale (CRS-r) [17] was lower than 8 in VS/UWS subjects and between 10 and 14 in the MCS. Subjects clinically unstable, under treatment with neuroactive drugs or beta-blockers or with concurrent systemic disorders or evidence of recurrent pain were not admitted to the study. Demographics and the relevant clinical information are summarized in Table 1.

**Table 1:** Summary of the patients’ demographics and clinical condition

**Vegetative state**						**Minimally conscious state**					
**Patient**	**Sex**	**CRS-R**	**Glasgow outcome**	**Etiology**	**Time from brain injury (yrs.)**	**Patient**	**Sex**	**CRS-R**	**Glasgow outcome**	**Etiology**	**Time from brain injury (yrs.)**
1	male	3	2	traumatic	5	1	male	13	3	traumatic	2
2		6	2	Nontraumatic	3	2		11	3	nontraumatic	4
3		6	2	nontraumatic	4	3		10	3	nontraumatic	3
4		5	2	traumatic	4	4		11	3	traumatic	3
5		7	2	traumatic	3	5		11	3	traumatic	4
6		5	2	traumatic	3	6		12	3	traumatic	5
7		6	2	traumatic	3	7		12	3	nontraumatic	5
8		7	2	traumatic	4	8		13	3	Traumatic	4
9		7	2	traumatic	5	9		13	3	Traumatic	3
10		5	2	nontraumatic	4	10	female	10	3	nontraumatic	4
11	female	6	2	nontraumatic	5	11		10	3	nontraumatic	4
12		5	2	nontraumatic	6	12		11	3	traumatic	4
13		5	2	traumatic	4	13		12	3	nontraumatic	5
14		6	2	traumatic	2	14		13	3	nontraumatic	3
						15		13	3	Traumatic	3
						16		12	3	Traumatic	3

The “Independent ethical committee ASP” of the public health administration of Crotone (Italy) approved the study and the experimental procedures. The ethical principles of the Declaration of Helsinki (1964) concerning human experimentation were carefully followed throughout the study. The patients’ relatives or caregivers were informed in full detail about the study and procedures and gave their written consent.

### Stimulus conditions and experimental procedure

Subjects were nursed before 9.00 am compliant to the unit rules and tested for a visual pursuit response at 9.30 – 10.30 am, i.e. at the time of the day when the response incidence had proven highest in a previous study [4]. They were comfortably sitting on armchair with constant 24°C ambient temperature in the absence of transient noise. A round mirror (12 cm in diameter) was moved slowly in front of the subjects in the horizontal and vertical planes for 45° (right, left, up, down) in order to obtain a visual pursuit response. The procedure was replicated in each direction and randomized. Subjects were tested when their eyes were open and no sign or sleep or drowsiness could be detected upon observation; in no case sensory or noxious stimuli were administered to stimulate patients into wakefulness. The test was performed by an expert neuropsychologist familial to all subjects; it was ranked positive for a consistent response when the patient’s eyes followed the mirror without loss of fixation. The procedure was in accordance with the CRS-r guidelines [17]. Nine (3.75%) of the 240 stimulus conditions were discarded because of artifact mostly due to sudden movements such as cough or grimace, with a total of 231 useful trials.

### Heart rate variability: data collection

The heart rate was recorded continuously before (5 min., baseline) and during each testing procedure by means of a photopletismograph positioned on the middle finger of the left hand and interfaced with a Nexus 10 commercial data acquisition system (http://www.mindmedia.nl); sampling was at 128 Hz, resolution at 24 bit, the average recording time during testing 224 ± 3 sec. These recording procedure and sampling rate were favored to minimize the subjects’ discomfort and the equipment interference; although not as precise as high-rate sampled EKG, it has proven reliable in studies on healthy [18] and in VS/UWS and MCS subjects [19] and is in agreement with the Task Force of European Society of Cardiology and the North American Society of Pacing and Electrophysiology guidelines [20]. The tachogram (*i.e.* the series of consecutive intervals between heart beats) was analyzed in the time and frequency domains by means of the Biotratace + (http://www.mindmedia.nl) and Kubios dedicated software for HRV measurements [21]. Non- parametric Fast Fourier Transform and Welch spectrum analyses were performed in the 0.01 to 0.5 Hz interval with 0.001 Hz resolution. The spectral descriptors in three frequency ranges (very low frequency [VLF]: 0.01–0.04 Hz; low frequency [LF]: 0.04–0.15 Hz; and high frequency [HF]: 0.15–0.5 Hz) were computed [20].

### Heart rate variability: selection of descriptors

Previous studies applying advanced data mining tools to sort out consistent trends or associations in large datasets [22,23] have identified the normalized LF index (*nuLF* ) and the LF peak (*pkLF*) as reliable descriptors of sympathetic/parasympathetic function in the VS/UWS and MCS [5,6,13,19]. These parameters are known to describe the control of cardiovascular function in adaptive behavior and the interaction between excitatory and inhibitory autonomic control mechanisms and were thus entered into the statistical analyses. Specifically, *nuLF* is deemed indicative of sympathovagal balance [24]. The 0.1 Hz component *LF* (pkLF) describe the sympathetic/parasympathetic interplay mediated by baroreflex and has been related to changes in arousal and to emotional responses [25].

### Descriptive statistics and modeling

The HRV descriptors of the VS/UWS and MCS subgroups were compared in baseline to exclude differences depending on the clinical condition already at rest (Mann–Whitney U test). The correlation of the presence/absence of a visual pursuit response with each HRV descriptor was also tested by the Mann–Whitney test. The probability of observing a visual pursuit response was estimated as the relative frequency of response for each subject vs. each HRV descriptor (*nuLF*, peak *pkLF*). The dataset was also analyzed by *feature selection* technique and *Support Vector Machine* classifier in order to model the probability of observing a visual pursuit response on the basis of the actual HRV measurements. Feature selection is used in predictive data techniques whereby relevant features (variables) are to be selected to build robust learning models and develop predictive models for the event of interest based on a large number of predictors [26]. Support Vector Machine (SVM) is a machine learning methodology of widespread use in classification, regression and ranking [27,28]. A state of art classification method with high accuracy and flexibility, SVM is used in bioinformatics and other disciplines to model data of varying source and meaning [28]; applicability in HRV measures processing has been documented [29]. In this study, SVM was used to develop a model able to predict target data values (presence or absence of a visual pursuit response) to which specific attributes (the HRV descriptors) could be related at the different steps of the training set for classification. The Generalization levels of correct classifications (model) were tested and validated by the Leave One Out and Ten Fold Cross Validation tests [30]. The Radial Basis Function (RBF) was selected as the kernel function to be used to solve the nonlinear problem [31]. The model *sensitivity* and *specificity* were estimated as the proportions of actual *presence* or *absence* of visual pursuit correctly classified. The method conditions of application allow reasonably exclude a “double dipping” bias due to the use of the same data for selection and selective analysis and possibly resulting in distorted descriptive statistics and invalid inference.

## Results

**Figure 1:**
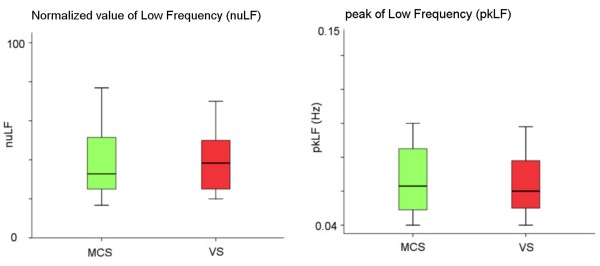
**Distribution of the HRV descriptors *nuLF *** **and *pkLF *** **in the VS/UWS (red) and MCS (green) patients’ subgroups at baseline.**

A visual pursuit response was observed in 33% and 78.2% of subjects in VS/UWS or MCS, respectively. The distributions of the HRV descriptors in the subgroups of VS/UWS or MCS subjects did not differ in baseline (Mann Whitney U test z > −0.665, p > 0.5) (Figure 1).

**Figure 2:**
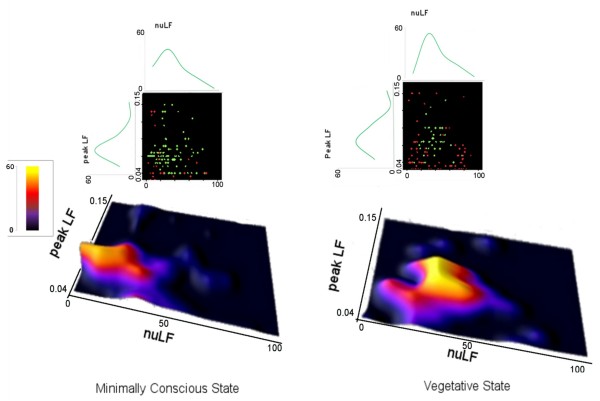
**Above, two-dimension distribution of positive (green) and negative visual pursuit response (red) in the VS and MCS patients versus the *nuLF *(abscissa) and *pkLF *values (ordinate) (distribution curves are also shown).** Below, probability of observing a positive pursuit response estimated as the relative frequency of response for each subject versus the HRV descriptors.

The observation of a visual pursuit response depended on the *nuLF* and *pkLF* values in both patients’ subgroups. The positive pursuit responses of the VS/UWS patients were clustered (76.6%) at *pkLF* values between 0.06 and 0.12 Hz and *nuLF* values between 20 and 60; in MCS, the positive responses were clustered (80.7%) at 0.05-0.10 Hz *pkLF* values and *nuLF* values between 10 and 60 (Figure 2). The estimated probability of observing a response was described by polynomial (cubic) curves, with a maximum peak of response in both VS/UWS and MCS subgroups at *nuLF* values between 20 and 40 (R^2^: 0.918 and 0.854 for the VS/UWS and MCS, respectively) and *pkLF* values at 0.06-0.08 Hz (R^2^: 0.999 and 0.937 for the VS/UWS and MCS, respectively) (Figure 2). The *pkLF* values interval at which positive responses clustered was comparable in the VS/UWS and MCS subgroups (χ^2^ = 2.811, p = 0.58), while that of *nuLF* was wider in the MCS subgroup (χ^2^ = 14.593, p = 0.005).

The SVM tool modeled a nonlinear function describing the relationship of the HRV descriptors *pkLF* and *nuLF* with the presence or absence of a pursuit response. In the training test, the SVM-generated model gave percentages of correct classification of 88.8% and 86.9% in MCS and VS subgroups, respectively (Table 2). The good correct classification was confirmed at both validation tests (Table 2).

**Table 2:** Correct classification of response in the visual pursuit

		**Correct classification**	**Accuracy**	**Balanced accuracy**	**Sensitivity**	**Specifity**
General Model	Training Test	81.4	81.4	81.3	82.1	80.4
	10 Fold Cross	81.4	81.4	81.3	82.1	80.4
	Leave One Out	81	79.5	77.9	84.4	71.4
MCS Model	Training Test	88.8	88.8	90.3	88.3	92.3
	10 Fold Cross	87.9	87.9	89.6	87.5	91.7
	Leave One Out	82	82.1	82.6	81.8	83.3
SV Model	Training Test	86.9	87	84.9	78.9	90.9
	10 Fold Cross	84.3	84.4	81.9	74.4	89.5
	Leave One Out	77	76.9	78.4	81.8	75

## Discussion

Converging neuroimaging, electrophysiological and clinical observations provide evidence of direct/indirect functional links between HRV measures, autonomic control, and brain activity in structures that are involved also in attention and conscious processes [32,33]. In this regards, a model network (The Central Autonomic Network; CAN) has been proposed, in which the anterior cingulate cortex and its projections to the prefrontal cortex, amigdala, hypothalamus and brainsteam are involved in the modulation of autonomic output in response to pain and emotional or behavioral stimulus conditions [13]. Neuroimaging studies in healthy volunteers have shown that the visual pursuit response depends on activation in structures of the anterior and posterior midline (mesiofrontal and precuneal cortices) [34] which are metabolically impaired in the brain damaged with severe disorder of consciousness who are incapable of sustained visual pursuit [35]. The re-appearance of a positive response in these subjects is thus regarded as indicative of functional upgrading in the subject’s clinical condition and substantial recuperation of the corticocortical and brainstem-cortex interaction that is thought to be anatomically/functionally interfered with in the VS/UWS and MCS [2,8,9,35,36]. The observed higher incidence of a pursuit response at values of HRV descriptors deemed indicative of sympathetic/parasympathetic balance suggests that its within-day incidence (and, by extension, the subject’s variability in brain responsiveness) can depend on the functional status of the autonomic system in VS and MCS subjects. This would also add to the existing controversy [35] on whether visual pursuit indicates “automatic” subcortical processing compatible with, but atypical for the VS/UWS [37], or it signals higher order cortical activation and partially recovered consciousness [38].

Independent factors modulating both the brain responsiveness and the autonomic balance are also possible. Residual circadian/ultradian cycles asynchronous among subjects are conceivable. Spontaneous fluctuations and end-effects of neuronal or nonneuronal variables known to individually or collectively account for individual variability [4,39] cannot be excluded in principle no matter how controlled the conditions of observation. Cohort studies on larger groups of subjects would supplement our observation and provide information on underestimated source of individual variability.

It should nevertheless be noted how the observed incidence of a visual pursuit response in VS/UWS and MCS subjects’ subgroups (32.2% and 79.3%, respectively) and its variability [4] are congruent with the estimated rate of misdiagnosis (30-40%) [3] between two conditions with somehow unclear boundaries, that share etiology and underlying pathophysiology, but differ as to prognosis, medical, legal, or popular perception of the bioethical issue, allocated resources, healthcare policies, etc. [1,40]. Due to its correlation with a positive visual pursuit response, the sympathetic/parasympathetic balance could also qualify as an independent prognostic index to the extent monitoring it can be noninvasive (as in the case of HRV measurements) and reliable. Research on the correlation between the sympathetic/parasympathetic balance and the subject’s responsiveness to simple sensory or noxious stimuli is in progress.

## Conclusions

Application of HRV descriptors in the functional characterization of patients with severe disorder of consciousness appears promising [5-7,13] for references. Accurate monitoring of the sympathovagal balance would allow test the visual fixation and eye pursuit response with greater accuracy and have these signs eventually reconsidered as indices of recovered consciousness or predictors of evolution and outcome. By extension, the autonomic system functional state should be re-considered as an independent variable potentially affecting other measures of CNS function of varying complexity, from clinical signs to indices of regional brain activation.

## Competing interests

The authors declare that they have no competing interests.

## Authors’ contributions

MDC and FR collected all the data. FR performed all data pre- processing and statistical analyses. All authors were involved in conceiving the study and participated in its design and coordination. All authors contributed to the manuscript from draft to its final version and have agreed on the submitted manuscript. All authors read and approved the final manuscript.

## Pre-publication history

The pre-publication history for this paper can be accessed here:

http://www.biomedcentral.com/1471-2377/13/164/prepub
